# Assessing the effects of a two-amino acid flexibility in the Hemagglutinin 220-loop receptor-binding domain on the fitness of Influenza A(H9N2) viruses

**DOI:** 10.1080/22221751.2021.1919566

**Published:** 2021-04-29

**Authors:** Yixue Sun, Yulin Cong, Haiying Yu, Zhuang Ding, Yanlong Cong

**Affiliations:** a Laboratory of Infectious Diseases, College of Veterinary Medicine, Key Laboratory of Zoonosis Research, Ministry of Education, Jilin University, Changchun, People’s Republic of China; b JilinResearch & Development Center of Biomedical Engineering, Chanchung University, Changchun, People's Republic of China

**Keywords:** Influenza, H9N2 subtype, 220-loop, site substitutions, receptor recognition, immune evasion, replication, transmissibility

## Abstract

The enzootic and zoonotic nature of H9N2 avian influenza viruses poses a persistent threat to the global poultry industry and public health. In particular, the emerging sublineage h9.4.2.5 of H9N2 viruses has drawn great attention. In this study, we determined the effects of the flexibility at residues 226 and 227 in the hemagglutinin on the receptor avidity and immune evasion of H9N2 viruses. The solid-phase direct binding assay showed that residue 226 plays a core role in the receptor preference of H9N2 viruses, while residue 227 affects the preference of the virus for a receptor. Consequently, each of these two successive residues can modulate the receptor avidity of H9N2 viruses and influence their potential of zoonotic infection. The antigenic map based on the cross-hemagglutination inhibition (HI) titers revealed that amino acid substitutions at positions 226 or 227 appear to be involved in antigenic drift, potentially resulting in the emergence of H9N2 immune evasion mutants. Further analysis suggested that increased receptor avidity facilitated by residue 226Q or 227M resulted in a reduction in the HI titer. Among the four naturally-occurring amino acid combinations comprising QQ, MQ, LQ, and LM, the number of viruses with LM accounted for 79.64% of the sublineage h9.4.2.5 and the rescued virus with LM exhibited absolute advantages of *in vitro* and *in vivo* replication and transmission. Collectively, these data demonstrate that residues 226 and 227 are under selective pressure and their synergistic regulation of receptor avidity and antigenicity is related to the evolution of circulating H9N2 viruses.

## Introduction

Avian influenza viruses (AIVs) belong to the genus *Alphainfluenzavirus* of the virus family *Orthomyxoviridae* [1]. They not only cause serious economic damage to the global poultry industry, but also occasionally pose substantial threats to human health [2]. H9N2 is one of the most widespread subtypes of the AIV family, which has been found in wild birds and as an enzootic pathogen in poultry across much of Asia and parts of Africa since 1966 [3]. For decades, H9N2 viruses have evolved to break through the interspecies barrier transmitting to pigs, dogs, horses, mink, pika, and bats [4,5]. It is noteworthy that the long-term prevalence favors the interspecies transmission of H9N2 viruses from avian species to humans. From July 1998 to August 2020, at least 69 people worldwide were infected with H9N2 viruses [5,6]. Retrospective serosurveys also revealed high seropositivity rates for H9N2 antibodies among poultry workers [5,6]. These findings indicate that H9N2 viruses have gained the capacity to overcome host restriction factors. The enzootic and zoonotic nature of H9N2 viruses is a great source of concern and emphasizes the importance of comprehensively understanding the fitness of this particular subtype of AIVs.

The hemagglutinin (HA) protein on the surface of influenza A viruses (IAVs) is a transmembrane homotrimer that functions actively through proteolytic cleavage of the HA precursor HA0 into two active subunits, HA1 and HA2. The specific binding of the HA1 globular head to the sialic acid (SA)-containing receptor on the target cell, which is the initial step in virus entry, determines the viral host range [5]. Human IAVs typically display preferential binding to SAα2,6Gal (human-type receptor), which is prevalent in the upper respiratory tract of humans [7], while avian-origin viruses bind preferentially to SAα2,3Gal (avian-type receptor), which is found throughout the avian respiratory and gastrointestinal tracts [8]. The receptor-binding property of IAVs depends on the receptor-binding domain (RBD) in the globular head of HA1. The shallow RBD pocket contains three secondary structural elements, comprising the 130-loop, 190-helix, and 220-loop [9]. The residues within the 220-loop (residues 221-228) (H3 numbering used throughout) are known to be the major determinants of the HA binding preference for either “human-type” or “avian-type” receptors [10].

Previous studies have shown that a single Q226L change plays a key role in receptor specificity switching and is thought to be associated with mammalian tropism of IAVs [11–13]. Furthermore, the Q226L substitution can significantly influence hemagglutination inhibition (HI) titers owing to changes in receptor avidity. For example, the L226Q substitution of H9N2 viruses reduced HI titers against antisera raised to either a 226Q or a 226L virus [3]. This indicates that the regulatory effect of residue 226 on the receptor avidity contributes to an increase in the chance of evading immune responses. Nevertheless, recent evidence suggests that residue 226 cannot be regarded as the only molecular marker of the biological characteristics of H9N2 viruses without considering the cooperative effect of surrounding amino acids, notably at position 227 [14,15]. Therefore, a more specific focus on the synergistic effects of the residues at positions 226 and 227 is required to fully understand the molecular basis of the correlation between viral receptor avidity and immune evasion. Here, we systematically evaluated the cooperative effects of four naturally-occurring variation combinations at residues 226 and 227 of H9N2 viruses from the perspective of receptor preference, cell avidity, antigenic variability, and replicative and transmission potentials. This information will clarify the genetic basis of the correlation between modulation of receptor avidity and change in antigenicity in order to assess the zoonotic potential and risk of immune evasion, as well as contributing to the rational design of vaccines against H9N2 viruses.

## Materials and methods

### Ethics statement

The protocols involving animal studies were performed in accordance with relevant guidelines and regulations made by the Committee on the Ethics of Animal Experiments of Jilin University.

### Diversity analysis of amino acid residues

The amino acid diversity across the HA 220-loop was calculated by the inverse Simpson index according to all available 9,392 HA sequences of H9N2 viruses from the NCBI influenza database (https://www.ncbi.nlm.nih.gov/genomes/FLU/Database/nph-select.cgi?go = database) and the GISAID EpiFlu database (http://platform.gisaid.org) as of September 1, 2020.

### Phylogenetic analysis

A phylogenetic tree of the full-length HA gene based on the maximum-likelihood method was constructed through the CIPRES Science Gateway (http://www.philo.org) using RaxML-HPC (v.8.2.10) [16]. The lineages and sublineages of HAs were divided as described previously [17].

### Analysis of selection pressure sites

The online Datamonkey server (http://classic.datamonkey.org/) was used to analyze the selection pressure sites of H9N2 HAs. In brief, the alignment results of multiple HA amino acid sequences were exported to FASTA format using Mega 7.0 software. After online submission, four different models including the single likelihood ancestor counting (SLAC), fixed effects likelihood (FEL), fast unbiased bayesian approximation (FUBAR), and mixed effects model of evolution (MEME) were selected to estimate the ratio of non-synonymous (dN) to synonymous (dS) substitution (dN/dS), ω, β-α, or ω^+^ per site.

### Cells

Primary chicken embryo fibroblast (CEF) cells were prepared from 9-day-old specific-pathogen-free (SPF) embryonated chicken embryos as described previously [18]. CEF, Madin-Darby canine kidney (MDCK) cells (ATCC, CCL-34), human embryonic kidney (HEK 293T) cells (ATCC, CRL-11268), and human lung adenocarcinoma (A549) cells (ATCC, CCL-185) were cultured in Dulbecco’s modified Eagle medium (DMEM) (Gibco, USA) supplemented with 10% fetal bovine serum (FBS) (Gibco, USA) and antibiotics at 37 °C with 5% CO_2_.

### Generation of reverse genetics viruses

The desired mutations were introduced into the HA gene (GenBank Accession No. KY785896) from A/quail/Hong Kong/G1/1997 (G1) by the site-directed mutagenesis kit (Qiagen, USA). The constructed genes encoding different naturally-occurring two-amino acids at 226 and 227 sites were cloned into a dual-promoter plasmid, pHW2000 [19]. All site-substitution viruses in this study were rescued through reverse genetics [20]. In brief, HEK 293 T cells grown in a 6-well plate were transiently co-transfected with 1 μg each of the seven plasmids of G1 virus plus the pHW2000-HA encoding variant HA proteins using TransIT®-293 (Mirus, USA). At 48 h post-transfection, the culture supernatants were inoculated into 9-day-old SPF embryonated chicken eggs (Beijing Merial Vital Laboratory Animal Technologies Co., LTD, Beijing, China). At 3 days post-inoculation (dpi), the harvested allantoic fluids were tittered by plaque assay (giving PFU per ml) on MDCK cells.

### Solid-phase direct binding assay

To directly examine the intrinsic receptor preference of H9N2 viruses, a solid-phase direct binding assay with a streptavidin–biotin detection system was used as described previously [21]. Four different receptor glycopolymer analogs were used: Neu5Acα2,3Galβ1,4Glc-PAA-biotin (3′SL) and Neu5Acα2,6Galβ1,4Glc-PAA-biotin (6′SL) were obtained from Glycotech, USA; Neu5Acα2,3Galβ1,4GlcNAc-SpNH-PAA-biotin (3′SLN) and Neu5Acα2,6Galβ1,4GlcNAc-SpNH-PAA-biotin (6′SLN) were kindly provided by the Consortium for Functional Glycomics, USA.

### Receptor avidity assay

The receptor avidity assay was performed according to the protocol as described by Peacock *et al*. [3]. Briefly, 1% chicken red blood cells (RBCs) were treated at 37 °C for 1 h with 2-fold serial dilutions of the receptor-destroying enzyme (RDE; neuraminidase from *Vibrio cholerae*) (Sigma-Aldrich, USA) to remove SAs. Subsequently, the RBCs were washed three times and prepared into a 1% suspension with PBS. Then, 50 μl of RDE-treated RBCs were incubated with an equal volume of a standard amount of each virus (4 HAU) on ice for 45 min to record the highest concentration of RDE that can allow complete hemagglutination.

### Generation of antisera

The antisera were prepared as described previously [22]. In brief, 3-week-old SPF white Leghorn chickens (Beijing Merial Vital Laboratory Animal Technologies Co., LTD, Beijing, China) were vaccinated intramuscularly (leg muscle) with 0.5 ml of 0.1% paraformaldehyde-inactivated viruses (64 HAU) in the presence of Montanide adjuvant. At 21 days post-vaccination, the collected sera were inactivated at 56 °C for 50 min and pretreated with RDE overnight at 37 °C to remove nonspecific inhibitors. The pooled sera were kept at −20 °C until use.

### Cross-HI assay

The HI assay was performed as previously described [23]. In brief, 8 HAU of each virus was added to serially 2-fold diluted sera. After incubation at 37 °C for 1 h, 50 μl of 1% RBCs were added. The homologous and heterologous HI titers were expressed as the reciprocal of the highest serum dilution that completely inhibited hemagglutination and converted into log_2_ values.

### Construction of virus antigenic map

The cross-HI dataset consisted of 12 antisera against 4 variants (3 chickens/variant). The result was expressed with a 12×4 matrix, where each row depicts the differences in HI titers between each of 4 variants against each of the 12 antisera. The virus antigenic map was constructed as previously described [24,25]. In brief, this 12×4 matrix was standardized so that the average of each virus against each of the 12 antisera would be 0 and the SD would be 1. Accordingly, we calculated the distances between variants by the following formula and obtained a resulting symmetrical 4×4 distance matrix.

$$dist=\sqrt{\sum_{k=1}^{12}(m_{i,j}-m_{i,j}{)}^{2}}$$

### Viral growth kinetics

The viral growth kinetics was expressed by one-step and multi-step growth curves in CEF, A549, and MDCK cells to identify the zoonotic infection potential and replication characteristics of these 4 variants. For the one-step growth curves, each virus at an MOI of 0.01 was inoculated into confluent monolayer cells. After 1 h of virus adsorption at 37 °C, cells were washed twice and overlaid with 2 ml DMEM containing 0.3% BSA, antibiotics, and 2.5 μg/ml of TPCK-treated trypsin. The supernatants of cell culture were sampled at 2 h intervals until 12 h post-inoculation (hpi). The viral TCID_50_ was determined by the method of Reed and Muench [26]. The multi-step growth curves were similarly conducted except with a starting inoculation at an MOI of 0.001.

### In vivo viral growth competitive and transmission experiment

The *in vivo* studies on the competitive properties of these 4variants were performed as described by Obadan *et al*. [12]. Three-week-old SPF chickens were randomly divided into 6 groups, with 6 chickens per group. In group 1, chickens were intranasally inoculated with 0.5 ml of 10^6^ TCID_50_/ml virus mix containing a homogenous mixture of 4 variants. In groups 2–5, chickens were inoculated intranasally with 0.5 ml of 10^6^ TCID_50_/ml each of variants. Group 6 served as the negative control inoculated intranasally with 0.5 ml PBS. At 1 dpi, 6 naive chickens were introduced into each group as contacts to determine transmission. Tracheal and cloacal swabs from each chicken were sampled until 14 dpi.

### High-throughput sequencing

The RNA of tracheal swab samples was extracted so as to complete the whole-genome sequencing. Subsequently, a one-step RT–PCR was performed for the whole-genome amplification using a PrimeScript^TM^ one-step RT–PCR kit (TaKaRa, Japan). Amplicon sequence libraries were prepared using an Ion Xpress Plus Fragment Library Kit (Thermo Fisher Scientific, USA). Barcoded libraries were sequenced on a high-throughput Illumina MiSeq platform in a paired-end 200-nucleotide run format.

### Quantification of virus infection and transmission

To assess virus shedding, the tracheal and cloacal swabs were inoculated into 9–11 days SPF embryonated eggs. After 3 dpi, RNA in the allantoic fluids was extracted using a QIAamp Viral RNA Mini Kit (Qiagen, USA). A one-step quantitative RT–PCR (qRT-PCR) using an SYBR® Green qRT-PCR kit (Sigma-Aldrich, USA) was carried out using one pair of primers specific for the matrix gene of AIVs (primer sequences available upon request). The cycle threshold (Ct) values of the strains to be tested were evaluated by the standard curve constructed.

### pH stability

H9N2 viruses with 128 HAU/50 μl were mixed with an equal volume of 100 mM acetate buffer (pH 4.0 and 5.0), 100 mM phosphate buffer (pH 6.0), or 100 mM neutral phosphate buffer (pH 7.0). After incubation at 37 °C for 10 min, viral titers were determined by hemagglutination assay [27].

### Thermostability assay

The thermostability assay was conducted as described previously [22]. In brief, 128 HAU/50 μl of H9N2 viruses were incubated at 50 °C for different periods of time, respectively. Subsequently, the heat-treated virus samples were quickly cooled to 4 °C and hemagglutination assays were performed in triplicate to determine changes in virus hemagglutination titers.

### Statistical analysis

Significance of difference between data was analyzed by One-Way or Two-Way ANOVA methods in GraphPad Prism 8.0 software. *P *> 0.05 means no significant difference (ns) and *P *< 0.05 is considered statistically significant (*f or *P *< 0.05; ** for *P *< 0.01; *** for *P *< 0.001; **** for *P *< 0.0001).

## Results

### Flexibility and selective pressure at residues 226 and 227 of H9N2 viruses

**Figure 1.**
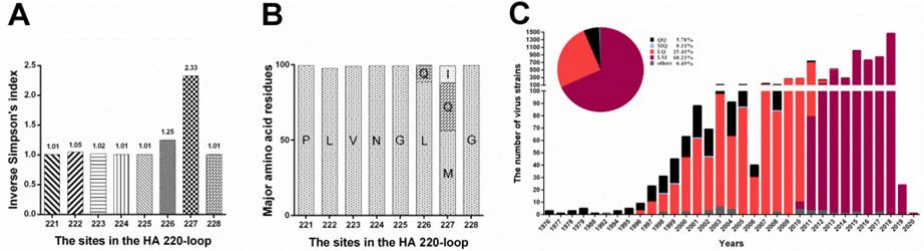
Analysis of amino acid diversity at residues 226 and 227 in the HA 220-loop of H9N2 viruses and the number changes of virus strains with different amino acid combinations at these two sites. In the 9,392 H9N2 virus strains worldwide, the amino acid diversity calculated by the inverse Simpson's index was indicated above each bar (A) and more than 10% of the major amino acids were showed in each bar (B). (C) The number changes of H9N2 virus strains with QQ, MQ, LQ, and LM at residues 226 and 227 isolated in China from 1976 to September 1, 2020.

The inverse Simpson index of the HA sequences from 9,392 H9N2 virus strains worldwide showed that the amino acid variation frequency of 226 and 227 sites were obviously higher than those of other sites within the 220-loop (Figure 1(A,B)). Further analysis indicated that residues 226 and 227 were under positive selection as detected by the models of SLAC, FEL, FUBAR, and MEME (Table S1). Among 9,392 H9N2 strains, the isolates in China (78.61%, n=7,383 of 9,392) had three major combinations at residues 226 and 227, consisting QQ (5.78%), LQ (25.41%), and LM (68.21%) (Table S2). From the perspective of epidemic time, however, the fitness of these strains in the poultry population has changed as shown in Figure 1C and Table S2, in which the viruses with LM became the predominant strains with an absolute epidemic advantage in recent years. Based on the phylogenetic tree of 6,512 H9N2 strains in China with the full-length HA genes (Figure S1), it was further revealed that the strains of more than 90.06% (n=5,865 of 6,512) belonged to the sublineage h9.4.2.5, while the strains of 79.67% (n=4,673 of 5,865) in this sublineage possessed LM (Table S3). All these findings promote us to evaluate the effects of these two successive amino acid residues on the fitness of H9N2 viruses.

### Receptor preference of H9N2 variants

**Figure 2.**
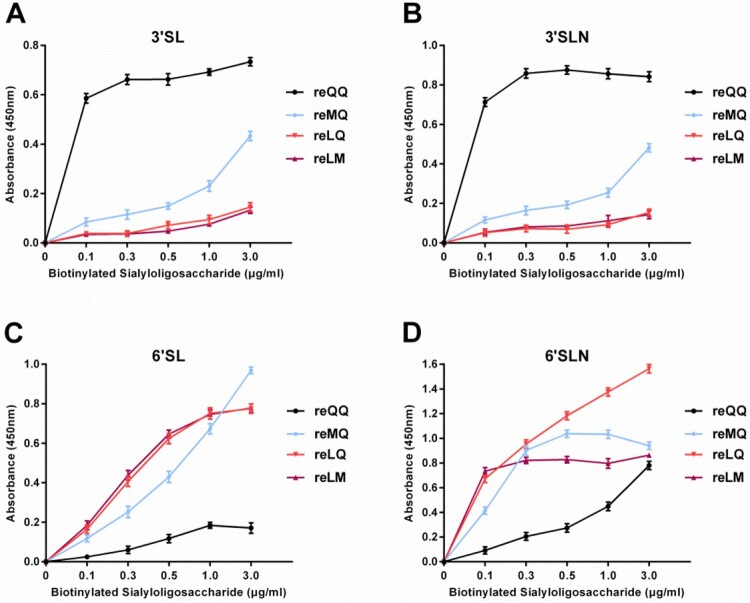
Receptor preference of H9N2 variants. Panels A, B, C, and D show the receptor preference determined by the solid-phase direct binding assays.

To investigate the synergistic effect of these two-amino acid substitutions at residues 226 and 227 on the receptor preference of H9N2 viruses, double site-substitutions consisting QQ, MQ, LQ, and LM were introduced into the HA gene of the G1 virus, which corresponded to the naturally-occurring variants of H9N2 viruses. The rescued four viruses were designated reQQ, reMQ, reLQ, and reLM, respectively. The solid-phase direct binding assay showed that reQQ exhibited a strong preferential binding to avian-type receptors such as 3'SL and 3'SLN (Figure 2(A,B)). When 226Q was replaced by L, however, those viruses with 226L, such as reLQ and reLM, bound preferentially to human-type receptors such as 6'SL and 6'SLN (Figure 2(C,D)). As for the Q226M substitution, it allowed reMQ to recognize both human-type and avian-type receptors, although a stronger preference for binding to the former was observed (Figure 2(A-D)). In general, the effects of amino acid substitutions suggest that residue 226 plays a central role in the receptor preference of H9N2 viruses, while residue 227 affects the preference of the virus to a receptor. As shown in Figure 2D, the preference of reLQ to 6'SLN was stronger than that of reLM.

### Relatedness between receptor avidity and antigenic difference of H9N2 variants

**Figure 3.**
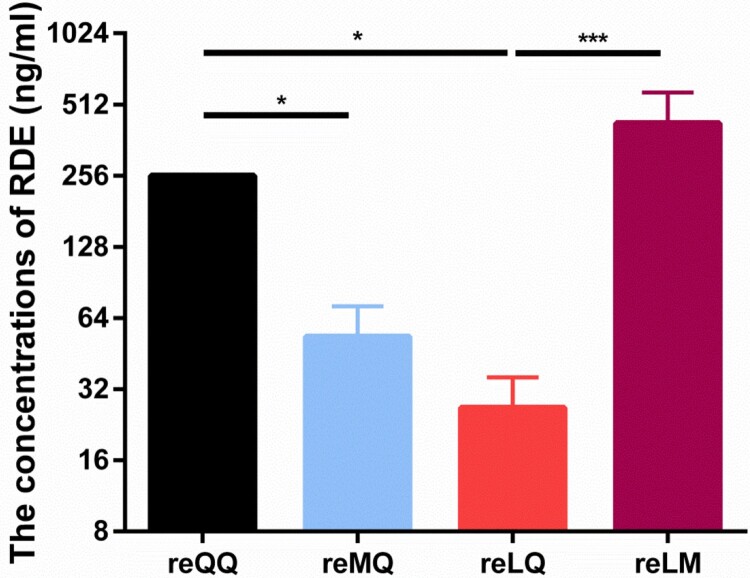
Receptor avidity of H9N2 variants. The erythrocyte-binding activity of H9N2 variants was determined by the cell avidity assays, where chicken red blood cells were desialylated by 2-fold serial dilutions (8-1,024 ng/ml) of RDE.

A previous study showed that the H9N2 viruses with 226Q exhibit higher receptor avidity for chicken RBCs than that of the 226L viruses, resulting in a general reduction in HI titers [3]. To confirm the influence of amino acid substitutions at positions 226 and 227 on the receptor avidity for RBCs, we carried out an RDE-based erythrocyte-binding assay to measure the ability of these four variants to agglutinate chicken RBCs treated with varying concentrates of RDE. The results showed that the hemagglutination activity of reMQ and reLQ was significantly decreased compared with that of reQQ (both *P* < 0.05). Conversely, the exchange of Q for M at residue 227 of reLQ resulted in a significant increase in the hemagglutination activity of reLM (*P *< 0.001) (Figure 3).

**Figure 4.**
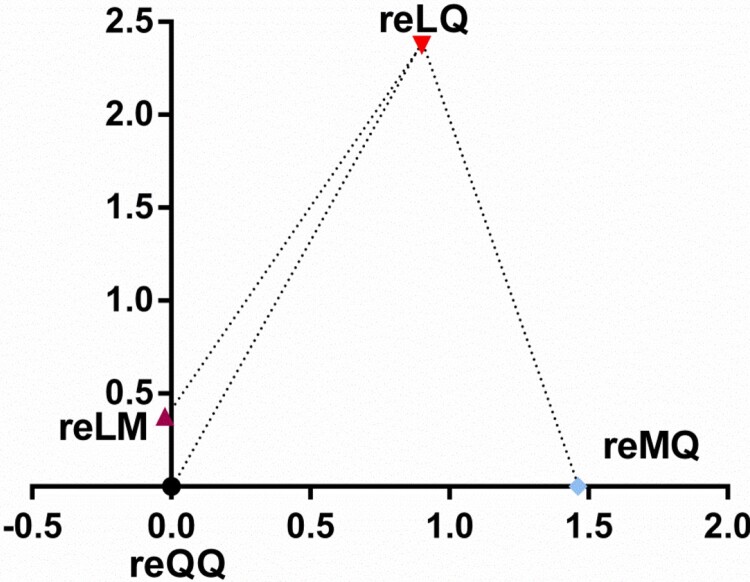
Antigenic map of H9N2 variants. Distances are represented by log_2_ values.

To explore the effect of these two amino acid substitutions at positions 226 and 227 on the relatedness between receptor avidity and antigenic difference of these four variants, we generated antisera in chickens and evaluated the magnitude of antigenic distances between these four variants by a cross-HI assay. As shown in Table 1, the Q226L substitution made the HI titers increase by 1 log_2_ to 2 log_2_. Similarly, the HI titers were increased by 2 log_2_ to 4 log_2_ due to the M226L substitution. However, the Q227M substitution generally reduced the HI titers by 1 log_2_ to 2 log_2_. To visualize the raw cross-HI data as a whole, the antigenic distances between the variants were calculated and then a simple and immediately visualized antigenic map was constructed as described previously [24,25]. As shown in Table S4 and Figure 4, the antigenic distances between reLQ and reQQ or reMQ were 2.54 or 2.44, respectively, while that between reQQ and reMQ was 1.46. In addition, there was an antigenic distance of 2.38 between reLM and reLQ. Collectively, these data demonstrate that the substitutions whether at residues 226 or 227 can affect the receptor avidity of H9N2 viruses and thus change their antigenicity.

**Table 1.** Cross-hemagglutination inhibition analysis of virus variants and antisera. A total of 12 antisera were prepared from four variants (3 sera/variant). Each antiserum is denoted by an animal serial number. Homologous titers are shown in bold.

Sera		Viruses			
Vaccine groups	Animal serial numbers	reQQ	reMQ	reLQ	reLM
reQQ	1	**64**	256	1,024	64
	2	**64**	512	1,024	32
	3	**64**	256	1,024	64
reMQ	4	16	**32**	512	16
	5	32	**64**	1,024	32
	6	16	**64**	1,024	16
reLQ	7	128	256	**512**	64
	8	128	256	**1,024**	64
	9	256	512	**1,024**	128
reLM	10	64	128	512	**64**
	11	64	128	512	**64**
	12	32	128	256	**64**

### In vitro and in vivo replicative fitness and transmission potential of H9N2 variants

**Figure 5.**
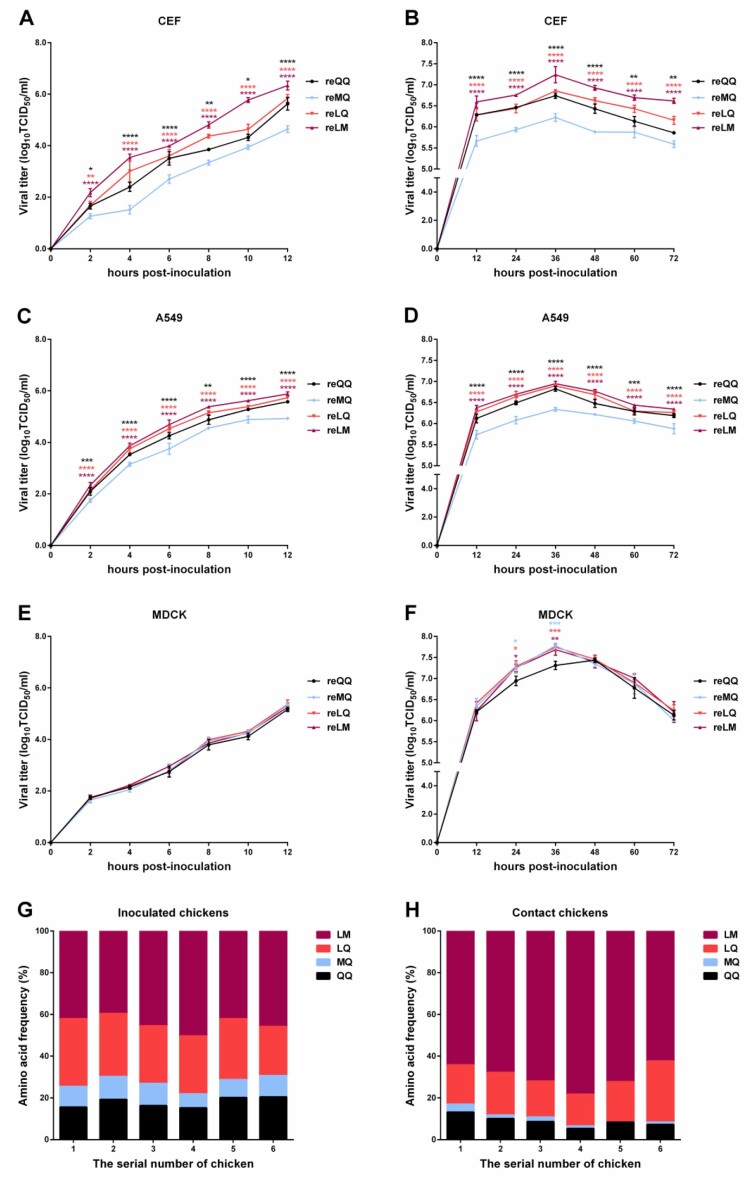
*In vitro* and *in vivo* growth dynamics of H9N2 variants. Panels A, C, and E show the one-step growth dynamic curves of H9N2 variants in CEF, A549, and MDCK cells, respectively. Panels B, D, and F show the multi-step growth dynamic curves of H9N2 variants in CEF, A549, and MDCK cells, respectively. At the indicated time points, virus titers in the supernatants were examined. Panels G and H show the frequency of the different HA sequences with specific amino acid residues at 226 and 227 in the tracheal swabs sampled from the inoculated chickens at 5 dpi (G) and from the contact chickens at 6 dpi (H). The sequences were obtained from NGS. The frequency of four different amino acid combinations was analyzed using Geneious 10.2.3 software. Each bar indicates a chicken in the respective group, and each color indicates a virus variant.

To assess the contribution of different amino acid combinations at positions 226 and 227 to cell tropism and replication of H9N2 viruses, we compared the dynamic growth curves of these four variants in different cells. As shown in Figure 5(A-F), all of these four variants can efficiently replicate in CEF, A549, and MDCK cells. However, the replication ability of reMQ in CEF cells (Figure 5(A,B)) and A549 cells (Figure 5(C,D)) was obviously lower than that of reQQ, reLQ, and reLM. Although the one-step growth curves showed no significant difference in the replication of these four variants in MDCK cells (*P *> 0.05) (Figure 5E), reQQ showed slightly lower replication attenuation at 24 and 36 hpi (*P *< 0.05-0.001) in the multi-step growth curves, compared with the other three variants (Figure 5F).

To ascertain which amino acid combinations at positions 226 and 227 were predominant and associated with contact transmission events, we determined the relative fitness advantage conferred by these four variants in an *in vivo* competitive model. Three-week-old chickens were intranasally inoculated with 0.5  ml of 10^6^ TCID_50_/ml containing a homogenous mixture of these four variants. The tracheal swabs sampled from the inoculated chickens at 5 dpi and from the contact chickens at 6 dpi were subjected to whole-genome sequencing by next-generation sequencing (NGS). According to the NGS sequencing results, we calculated the percentage of the different HA sequences with specific amino acid residues at 226 and 227 in all the obtained gene sequences. In detail, reQQ and reMQ were mapped to those sequences containing Q and M at residue 226, while those containing Q and M at residue 227 corresponded to reLQ and reLM, respectively. As a result, reLM occupied the predominance (39%-50%), followed by reLQ (23%-32%), reQQ (15%-20%), and reMQ (6%-10%) (Figure 5G). Similarly, there was a similar trend in the contact chickens, in which reLM, reLQ, reQQ, and reMQ accounted for 62%-78%, 15%-29%, 5%-13%, and 0%-3%, respectively (Figure 5H).

Subsequently, we investigated the comparative infectivity and transmissibility of these four variants in chickens. The clinical signs of disease were monitored daily. During the 14-d observation period, the inoculated chickens exhibited mild clinical signs, such as mild depression, anorexia, respiratory distress, and reduced activity levels, peaking between 4 and 6 dpi. In contrast, the contact chickens only showed low activity levels. Virus titration of tracheal and cloacal swab samples was evaluated by quantifying the RNA levels by qRT-PCR. The results showed that viral RNA in the tracheal and cloacal swabs was detected with a peak between 4 and 8 dpi in the inoculated chickens, while the peak for the contact chickens occurred between 6 and 8 dpi (Table 2). Whether in the inoculated chickens or in the contact chickens, reLM and reMQ showed the strongest and weakest infectivity and transmissibility in chickens, respectively. The results of this animal experiment well confirmed the epidemic potential of these four variants.

**Table 2.** Infection and transmission phenotype of H9N2 variants in the tracheal and cloacal swabs from inoculated and contact chickens. Viral shedding was quantified by qRT-PCR.

Samples	Viruses	Inoculated chickens (dpi)							Contact chickens (dpi)						
		2	4	6	8	10	12	14	2	4	6	8	10	12	14
Tracheal swabs	reQQ	2/6	5/6	6/6	6/6	3/6	2/6	2/6	0/6	1/6	4/6	4/6	2/6	1/6	1/6
	reMQ	0/6	1/6	2/6	2/6	1/6	1/6	0/6	0/6	0/6	1/6	1/6	0/6	0/6	0/6
	reLQ	3/6	4/6	6/6	6/6	4/6	4/6	3/6	0/6	3/6	4/6	5/6	4/6	2/6	2/6
	reLM	4/6	6/6	6/6	6/6	5/6	5/6	5/6	0/6	4/6	5/6	5/6	4/6	4/6	3/6
Cloacal swabs	reQQ	0/6	2/6	4/6	3/6	2/6	1/6	1/6	0/6	1/6	2/6	2/6	1/6	0/6	0/6
	reMQ	0/6	1/6	1/6	2/6	1/6	0/6	0/6	0/6	0/6	0/6	1/6	0/6	0/6	0/6
	reLQ	0/6	2/6	4/6	5/6	2/6	1/6	1/6	0/6	1/6	2/6	3/6	2/6	1/6	0/6
	reLM	1/6	3/6	5/6	5/6	3/6	3/6	3/6	0/6	2/6	3/6	4/6	2/6	1/6	1/6

### The pH and thermal stability of H9N2 variants

**Figure 6.**
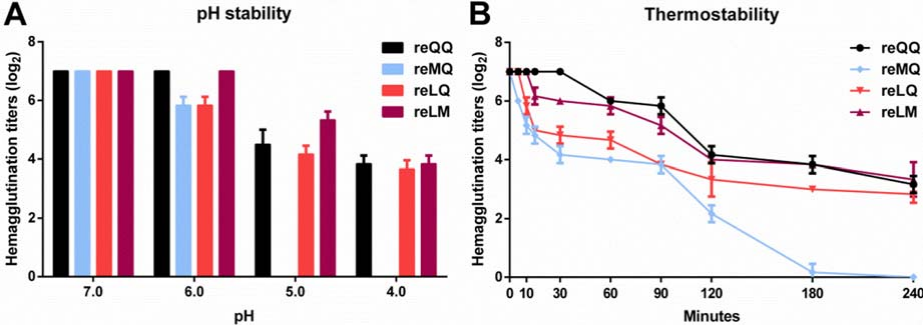
The pH and thermal stability of H9N2 variants. Each of these four variant viruses (128 HAU/50 μl) was exposed to variations in pH (A) or heated to 50 °C for different periods of time (B). The samples collected after incubation were analyzed in triplicate. The results are presented as log_2_ HA titers at the indicated pH conditions or time.

Previous studies have shown that substitutions in the RBD can change the pH stability of H5N1 and H7N9 viruses [28,29]. In this study, all these four variants were exposed to solutions adjusted to a range of pH values for 10 min to assess their pH stability. As shown in Figure 6A, the hemagglutination titers of these four variants were unchanged when exposed to a pH of 7.0. At pH 5.0 and 4.0, however, their hemagglutination titers were decreased obviously, where reMQ even completely lost its ability to agglutinate RBCs.

A correlation has been identified between pH stability and thermostability (Spearman’s rho=0.92) [22]. To compare the thermostability of these four variants, they were incubated at 50°C and the hemagglutination titers of samples collected at regular time-points during the 4-h time-course were determined. As shown in Figure 6B, all of them displayed decreasing thermostability after incubation at 50°C, although reQQ, reLQ, and reLM retained hemagglutination titers ≥2.5 log_2_ until 240 min. In contrast, the hemagglutination titers of reMQ decreased by between 2- and 8-fold before 180 min, and its hemagglutination activity was completely abolished at 240 min, suggesting that reMQ is the least stable.

## Discussion

A variety of factors determine whether IAVs can infect a host, including the recognition and binding of cell receptors, overcoming innate immunity, and evading the pre-existing immunity [30]. The HA protein of IAVs plays a critical role in these processes, given that it is responsible for the receptor and immunity recognition functions [31]. It is well-established that a single amino acid substitution at 226 site in the 220-loop of RBD is thought to be decisive in receptor specificity switching and associated with mammalian tropism of H9N2 viruses [11–13]. However, recent evidence suggests that residue 226 may not be the sole marker of the receptor preference of H9N2 viruses, without taking into consideration other amino acids in the vicinity [14,15]. By comparing the usage frequency of amino acids in the HA 220-loop of H9N2 viruses, we found that the amino acid variation frequency of 226 and 227 sites are relatively higher than those of other sites (Figure 1A). These two amino acids were mainly present in three combinations: QQ, LQ, and LM (Table S2). According to the number and epidemic time of H9N2 virus strains with these three kinds of amino acid combinations, it revealed that the QQ and LQ viruses had been gradually replaced by the emerging LM viruses since 2011 (Figure 1C and Table S2). However, the effect of different amino acid combinations at positions 226 and 227 on the fitness of H9N2 viruses remained to be clarified. In this study, we rescued four H9N2 variant strains possessing naturally-occurring amino acid combinations at residues 226 and 227. Among them, the purpose of rescuing a virus strain with MQ was to compare the biological effects of the residue 226 substitution.

The molecular basis of receptor binding has revealed that the amino acid substitutions at positions 226 and 227 can influence the spatial conformation of RBD, which in turn modulates the preference of viral HA for its receptor [12,13]. In accordance with other studies [17–19,32], our results also clearly demonstrated the decisive contribution of residue 226 in the receptor preference of H9N2 viruses. Meanwhile, we found that the substitution at residue 227 can influence the preference of the virus for a receptor, as shown by the obvious difference in the binding of reLQ and reLM to 6′SLN (Figure 2D). However, it remains to be clarified how the effect of the substitutions at residues 226 and 227 on the receptor preference further affects tissue tropism and host range of H9N2 viruses. Based on the 32 H9N2 virus strains isolated from humans registered in the NCBI influenza database and the GISAID EpiFlu database so far, we found that most of them (above 90%, n=29 of 32) had L at residue 226 (Table S5), indicating that the 226L viruses may have the great potential of zoonotic infection. In this study, the growth curves showed that the replication ability of these four variants in CEF and A549 cells demonstrated a trend of reLM > reLQ > reQQ > reMQ (Figure 5(A-D)), which further suggests that residue 226 has an obvious advantage over residue 227 in determining the infection potential of H9N2 viruses, although residue 227 also plays a role in this process.

Because of the persistent threat to poultry posed by H9N2 viruses, vaccination of poultry is a major preventive measure used to control H9N2 outbreaks in some enzootic countries including China, Egypt, Iran, Israel, Korea, Morocco, Pakistan, South and the United Arab Emirates [5]. Historically, the inactivated vaccines derived from some earlier strains with either QQ or LQ (Table S6) played an essential role in controlling H9N2 infections in mainland China. For example, the QQ and LQ viruses have been seldom isolated since 2017 and 2015, respectively (Figure 1C and Table S2). However, as with human seasonal IAVs, H9N2 viruses can undergo antigenic drift under the pressure of herd immunity, the consequence of which is the possible generation of novel immune evasion mutants. This is evidenced by the continued isolation of H9N2 viruses from all kinds of vaccinated poultry populations, especially the emerging and predominant sublineage h9.4.2.5 of H9N2 viruses in recent years [17]. It indicates that this specific sublineage of viruses may be immune evasion mutants. Previous studies have shown that amino acid substitutions within or around the RBD, which can alter epitope structure and thus directly affect antibody binding, are the most important mechanism of immune evasion [3,32,33]. Therefore, it is hypothesized that modulation of receptor avidity is a true form of immune evasion [3,34]. As the receptor-binding sites in the 220-loop RBD, positions 226 and 227 also have been identified as H9N2 antigenic sites [22]. Nevertheless, it remains unknown what effect these two amino acid substitutions will have on the relatedness between receptor avidity and immune evasion of H9N2 viruses. In this study, we confirmed that the Q/M226L substitution decreased the receptor avidity of H9N2 viruses, but increased the HI titers. As for the Q227M substitution, the correlation between receptor avidity and HI titers is the reverse (Figure 3 and Table 1). All these findings indicate that increased receptor avidity facilitated by the substitutions at residues 226 and 227 is one of the most important strategies of H9N2 viruses to evade immune. In addition, the antigenic map based on the cross-HI titers also indicates that these successive amino acid substitutions at positions 226 and 227 are involved in antigenic drift (Figure 4), potentially playing a role in the emergence of H9N2 immune evasion mutants. Combined with the lesser effectiveness of the contemporary vaccines against the LM variant strains and the epidemic dynamics of H9N2 viruses with different amino acid mutations at 226 and 227, it highlights the necessity of updating the H9N2 vaccine prototype with the current LM viruses in circulation. In addition to amino acid substitutions within or around the RBD and antigenic sites, however, it should be pointed out that the number change of glycans on HA is also an important molecular mechanism of the emergence of H9N2 immune evasion strains [3,10]. During screening for new H9N2 vaccine strains in future, these factors should be taken into full consideration to ensure that the vaccine preparations are up-to-date with the currently circulating strains, thus making the vaccines as effective as possible.

In nature, those airborne transmissible viruses that can generate adaptive genetic mutations under selective pressure, accompanied with such biological characteristics as high replication efficiency, low virulence and pathogenicity, and a broad host spectrum, may be more likely to survive. The low pathogenic H9N2 viruses, especially those viruses with LM, are a good example. In this study, we evaluated the contribution of various amino acid combinations at positions 226 and 227 to viral replication and transmission by both *in vivo* and *in vitro* experiments. High-throughput sequencing revealed that reLM had an absolute advantage of replication and transmission in both the inoculated and the contact chickens as well as in the single virus infection group and the virus mixed infection groups (Figure 5(G,H) and Table 2). The one-step and multiple-step growth kinetics curves also showed this obvious replication advantage of reLM among these four variants (Figure 5(A-D)). In contrast, reMQ exhibited the weakest replication and transmission capability, which could be related to the poor stability of reMQ in response to changes in pH and temperature (Figure 6(A,B)). It thus indicates that residue 226M may be a poor mutation of H9N2 viruses. All these findings may explain why the MQ viruses are rarely isolated in nature, while the QQ, LQ, and LM viruses thus have become the predominant strains for a time.

In conclusion, our study demonstrates that the synergistic substitutions at residues 226 and 227 can influence the fitness of H9N2 viruses as summarized in Table S7, especially contributing to the modulation of receptor recognition and antigenicity of H9N2 viruses. Furthermore, the regulation of receptor avidity is likely to be one of the important mechanisms for H9N2 viruses to evade immunity. Therefore, these two successive amino acid combinations can be considered as an adaptive mutation of H9N2 viruses. However, there are still some limitations in this study. For instance, it would be ideally performed in ferrets to assess the fitness of these four variants. Furthermore, a mixed system where ferrets and chickens are cohoused could provide some more interesting data on the effect of these two amino acid changes at positions 226 and 227 on the zoonotic potential of H9N2 viruses. In addition, it will be more convincing if we detect the effect of these two residue substitutions on the immune evasion of H9N2 viruses through the *in vivo* and *in vitro* neutralization experiments. Overall, a comprehensive study of the correlation between modulation of receptor avidity and change in antigenicity of this type is worthy of further in-depth and detailed exploration. This will help explain the fitness of H9N2 viruses in nature, as well as contributing to future vaccine design.

## Supplementary Material

Clean_copy_supplementary.docx
