# Supplementary material for: Assessing the effects of a two-amino acid flexibility in the Hemagglutinin 220-loop receptor-binding domain on the fitness of Influenza A(H9N2) viruses
Source: Emerg Microbes Infect. 2021 Apr 29;10(1):822–32. doi: 10.1080/22221751.2021.1919566 (PMC8812783; doi:10.1080/22221751.2021.1919566)
Supplement: Clean_copy_supplementary.docx [file TEMI_A_1919566_SM8779.docx]

## Supplementary file

**Table S1. Positive selection pressure sites of the HA proteins of H9N2 viruses analyzed by the SLAC, FEL, FUBAR, and MEME models.**

| Positions | SLAC | |  | FEL | |  | FUBAR | |  | MEME | |
| --- | --- | --- | --- | --- | --- | --- | --- | --- | --- | --- | --- |
|  | dN-dS | p-value |  | ω | p-value |  | β-α | Post. Pro β>α |  | ω^+^ | p-value |
| 137 | 1.78 | 0.0153 |  | 3.613 | 0.041 |  | 2.090 | 0.994 |  | >100 | 0.00 |
| 226 | 3.12 | 0.0116 |  | 2.090 | 0.009 |  | 4.307 | 0.999 |  | >100 | 0.00 |
| 227 | 3.64 | 0.0011 |  | 5.899 | 0.000 |  | 6.119 | 9.998 |  | >100 | 0.00 |

Note: Only the positive selection sites supported by the four detection models SLAC, FEL, FUBAR, and MEME are shown. The p-value of FEL and MEME models, SLAC model, and FUBAR model should be less than 0.05, 0.1, and 0.9 respectively.

**Table S2. Amino acid combinations at positions 226 and 227 of 7,383 H9N2 virus strains isolated in China from 1976 to September 1, 2020.**

| Positions | | The no. of isolates | The percentage of isolates (%) | Year of first isolation | Year of last isolation | Years of centralized isolation |
| --- | --- | --- | --- | --- | --- | --- |
| 226 | 227 |  |  |  |  |  |
| Q | Q | 427 | 5.78 | 1976 | 2017 | 1997-2014 |
| M | Q | 8 | 0.11 | 1998 | 2011 | 1998-2011 |
| L | Q | 1,876 | 25.41 | 1996 | 2015 | 1997-2013 |
| L | M | 5,036 | 68.21 | 2010 | 2020 | 2011-2019 |

**Table S3. Amino acid combinations at positions 226 and 227 of 6,512 H9N2 virus strains isolated from China.**

| Lineages | Sublineages | Total no. | Years of isolation (no.) |  | Residues at 226 and 227 (no.) |  | Geographical distribution (no.) |
| --- | --- | --- | --- | --- | --- | --- | --- |
| h9.1 | - | 10 | 1998 (1), 1999 (1), 2000 (2), 2001 (3), 2002 (1), 2008 (1), 2012 (1) |  | LQ (2), QQ (8) |  | Northeast (3), North China (1), East China (2), South China (2), Northwest (1), Southwest (1) |
| h9.2 | - | 3 | 1978 (2), 1979 (1） |  | QQ (3) |  | South China (3) |
| h9.3 | h9.3.1.1 | 1 | 1979 (1) |  | QQ (1) |  | South China (1) |
|  | h9.3.1.2 | 0 | - |  | - |  | - |
|  | h9.3.1.3 | 24 | 2011 (7), 2012 (16), 2017 (1) |  | QQ (24) |  | Central China (24) |
|  | h9.3.2 | 2 | 1988 (1), 1992 (1) |  | HK (1), QQ (1) |  | Southern China (2) |
|  | h9.3.3.1 | 0 | - |  | - |  | - |
|  | h9.3.3.2 | 2 | 1997 (2) |  | QQ (2) |  | Southern China (2) |
|  | h9.3.3.3 | 0 | - |  | - |  | - |
|  | h9.3.3.4 | 16 | 2004 (2), 2009 (1), 2011 (1), 2013 (1), 2014 (11) |  | QQ (16) |  | Northeast (1), East China (10), Southern China (2), Central China (2), Southwest (1) |
| h9.4 | h9.4.1.1 | 3 | 1997 (3) |  | LQ (3) |  | South China (3) |
|  | h9.4.1.2 | 58 | 1999 (3), 2005 (6), 2007 (1), 2009 (2), 2010 (1), 2011 (18), 2013 (7), 2014 (13), 2015 (6), 2017 (1) |  | HM (1), LQ (3), QQ (54) |  | East China (19), Southern China (39) |
|  | h9.4.1.3 | 0 | - |  | - |  | - |
|  | h9.4.1.4 | 0 | - |  | - |  | - |
|  | h9.4.1.5 | 0 | - |  | - |  | - |
|  | h9.4.2.1 | 59 | 1998 (5), 1999 (8), 2000 (4), 2001 (6), 2002 (8), 2003 (4), 2004 (2), 2005 (1), 2007 (1), 2008 (4), 2010 (1), 2011 (2), 2012 (4), 2013 (6), 2016 (1), 2017 (2) |  | LQ (7), QQ (52) |  | Northeast (1), North China (6), East China (43), Southern China (2), Central China (4), Northwest (2), Southwest (1) |
|  | h9.4.2.2 | 81 | 1998 (1), 1999 (1), 2000 (7)2001, (7) 2002 (11), 2003 (24), 2004 (22), 2005 (8) |  | LL (2), LQ (68), MQ (3), QQ (8) |  | East China (1), Southern China (77), Central China (2) |
|  | h9.4.2.3 | 23 | 1996 (5), 1997 (4), 1998 (1), 1999 (3), 2001 (3), 2003 (4), 2004 (1), 2007 (1), 2008 (1) |  | LQ (2), QQ (21) |  | Northeast (1), North China (5), East China (14), Southern China (2), Southwest (1) |
|  | h9.4.2.4 | 70 | 1997 (2), 1998 (7), 1999 (6), 2000 (11), 2001 (6), 2002 (4), 2003 (4), 2004 (5), 2005 (5), 2006 (2), 2007 (2), 2008 (4), 2009 (2), 2010 (1), 2012 (9) |  | LQ (33), QQ (37) |  | Northeast (12), North China (16), East China (13), Southern China (15), Central China (11), Northwest (2), China (1) |
|  | h9.4.2.5 | 5,865 | 1998 (3), 1999 (2), 2000 (15), 2001 (27), 2002 (21), 2003 (36), 2004 (21), 2005 (31), 2006 (18), 2007 (79), 2008 (63), 2009 (105), 2010 (238), 2011 (546), 2012 (161), 2013 (496), 2014 (256), 2015 (758), 2016 (759), 2017 (763), 2018 (1,442), 2019 (24), 2020 (1) |  | FQ (3), LE (1), LH (2) LI (1), LL (2), LM (4,673), LN (1), LQ (1,125), LR (3), MQ (2), QH (1), QM (1), QQ (50) |  | Northeast (54), North China (151), East China (2,945), Southern China (914), Central China (665), Northwest (267), Southwest (863), China (6) |
|  | h9.4.2.6 | 295 | 1996 (1), 1999 (1), 2000 (4), 2001 (4), 2002 (3), 2003 (18), 2004 (24), 2005 (32), 2006 (13), 2007 (28), 2008 (10), 2009 (5), 2010 (16), 2011 (91), 2012 (36), 2013 (5), 2014 (2), 2015 (1), 2016 (1) |  | LL (1), LQ (253), QQ (40), QR (1) |  | Northeast (7), North China (1), East China (37), Southern China (206), Central China (3)Northwest (4), Southwest（37） |

**Table S4. Virus distance matrix used for construction of the map of H9N2 variants.** A distance of 1 represents a difference value of 2 units in the HI assay because all distances are log_2_ values.

| Viruses | reQQ | reMQ | reLQ | reLM |
| --- | --- | --- | --- | --- |
| reQQ | 0 |  |  |  |
| reMQ | 1.46 | 0 |  |  |
| reLQ | 2.54 | 2.44 | 0 |  |
| reLM | - | - | 2.38 | 0 |

“-”, no comparison.

**Table S5. The amino acid combinations at positions 226 and 227 of human H9N2 virus strains isolated from China** **as of September 1, 2020.**

| Accession No. | Strains | Positions | | The no. of strains with this amino acid combination |
| --- | --- | --- | --- | --- |
|  |  | 226 | 227 |  |
| KM455872 | A/Lengshuitan/11197/2013 | L | M | 16 |
| EPIISL153064 | A/Hong Kong/308/2014 | . | . |  |
| KX595338 | A/Hunan/44557/2015 | . | . |  |
| EPIISL203644 | A/Hunan/44558/2015 | . | . |  |
| KU217316 | A/Zhongshan/201501/2015 | . | . |  |
| MF440728 | A/Beijing/1/2016 | . | . |  |
| KX808588 | A/Guangdong/MZ058/2016 | . | . |  |
| MF440736 | A/Beijing/1/2017 | . | . |  |
| EPIISL337279 | A/Hunan/42088/2017 | . | . |  |
| EPIISL330737 | A/Anhui-Lujiang/39/2018 | . | . |  |
| EPIISL337277 | A/Guangdong/18SF003/2018 | . | . |  |
| EPIISL345234 | A/Guangdong/18SF064/2018 | . | . |  |
| EPIISL345235 | A/Guangxi-Xiangshan/11522/2018 | . | . |  |
| EPIISL345236 | A/Hunan/34179/2018 | . | . |  |
| EPIISL407978 | A/Fujian-Sanyuan/2881/2019 | . | . |  |
| EPIISL410990 | A/Hong Kong/623/2020 | . | . |  |
| AY043015 | A/Shantou/239/98 | . | Q | 13 |
| AY043017 | A/Shaoguan/408/98 | . | Q |  |
| AY043018 | A/Shaoguan/447/98 | . | Q |  |
| AJ404626 | A/Hong Kong/1073/99 | . | Q |  |
| GU053179 | A/Hong Kong/1074/1999 | . | Q |  |
| KF188234 | A/Nanchang/CH2/2000 | . | Q |  |
| KF188272 | A/Nanchang/CH3/2000 | . | Q |  |
| KF188327 | A/Nanchang/D1/2000 | . | Q |  |
| KF188242 | A/Nanchang/D2/2000 | . | Q |  |
| DQ226106 | A/HK/2108/2003 | . | Q |  |
| KX867849 | A/Guangdong/W1/2004 | . | Q |  |
| CY055156 | A/Hong Kong/3239/2008 | . | Q |  |
| KF188289 | A/Hong Kong/69955/2008 | . | Q |  |
| KF188316 | A/Hong Kong/33982/2009 | Q | Q | 2 |
| CY055148 | A/Hong Kong/35820/2009 | Q | Q |  |
| AY043019 | A/Guangzhou/333/99 | M | Q | 1 |

Dots indicate residues identical to those of the A/Lengshuitan/11197/2013 virus.

**Table S6. The amino acids present at positions 226 and 227 of H9N2 vaccine strains previously or currently use in China.**

| Vaccine strains | Accession No. | Positions | |
| --- | --- | --- | --- |
|  |  | 226 | 227 |
| A/Chicken/Guangdong/SS/94 | AF384557 | Q | Q |
| A/chicken/Shandong/6/96 | DQ064376 | . | . |
| A/Chicken/Shanghai/F/98 | AY743216 | . | . |
| A/chicken/Jiangsu/1/1999 | FJ793388 | . | . |
| A/duck/Nanjing/01/1999 | DQ681221 | . | . |
| A/chicken/Shandong/1/02 | AY594196 | . | . |
| A/chicken/Guangxi/10/99 | DQ064363 | L | . |
| A/chicken/Shandong/S2/2005 | HM773440 | L | . |

Dots indicate residues identical to those of the A/Chicken/Guangdong/SS/94 virus.

**Table S7. Summary of the biological effects of four different amino acid combinations in the HA 220-loop of H9N2 viruses.**

| The biological phenotypes | | | | Residue combinations | | | |
| --- | --- | --- | --- | --- | --- | --- | --- |
|  |  |  |  | QQ | MQ | LQ | LM |
| Receptor preference | | Avian-type receptors | 3'SL | +++ | ++ | + | + |
|  |  |  | 3'SLN | +++ | ++ | + | + |
|  |  | Human-type receptors | 6'SL | + | +++ | +++ | +++ |
|  |  |  | 6'SLN | ++ | +++ | ++++ | ++ |
| Receptor avidity (Hemagglutination activity) | | | | +++ | ++ | + | ++++ |
| Antigenic distance (log_2_) | | | | 0 | 1.46^a^ | 2.54^a^ | 2.38^b^ |
| Proliferation fitness | *In vitro* replicative fitness | One-step growth dynamics (12 hpi) | CEF | +++ | + | +++ | +++ |
|  |  |  | A549 | +++ | + | +++ | +++ |
|  |  |  | MDCK | ++ | ++ | ++ | ++ |
|  |  | Multi-step growth dynamics (36 hpi) | CEF | ++ | +++ | +++ | ++++ |
|  |  |  | A549 | ++ | +++ | +++ | +++ |
|  |  |  | MDCK | +++ | ++++ | ++++ | ++++ |
|  | *In vivo* proliferation advantage | | | ++ | + | +++ | ++++ |
| *In vivo* transmission phenotype | | | | ++ | + | +++ | ++++ |
| pH stability | | | | +++ | + | +++ | ++++ |
| Thermal stability | | | | ++++ | + | ++ | ++++ |

^a^ represents the antigen distance from reQQ.

^b^ represents the antigen distance from reLQ


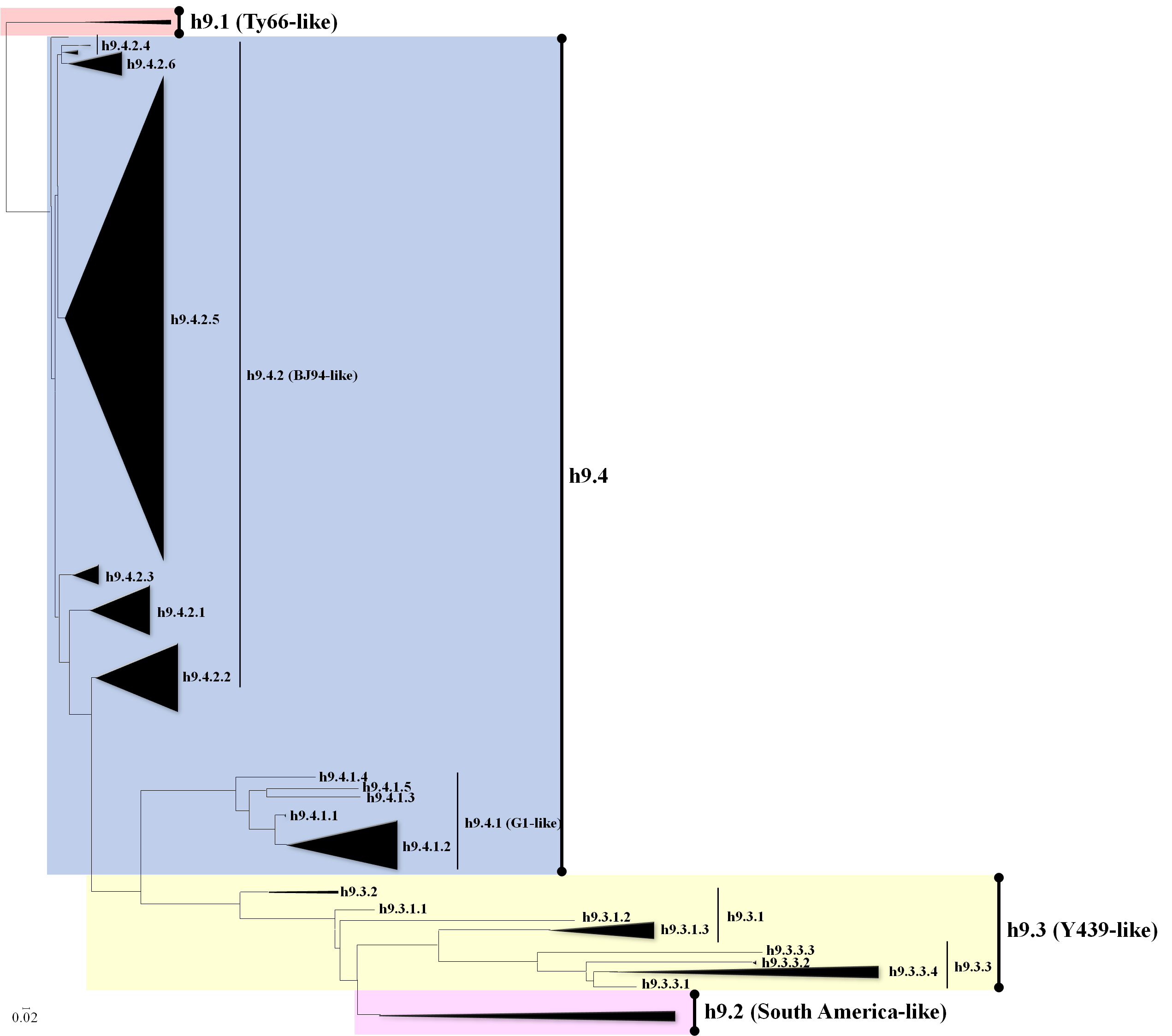


**Figure S1**

## Figure caption

**Figure S1. The phylogenetic tree based on the HA genes of H9N2 isolates in China (n=6,512).** The evolutionary history was inferred by using RaxML and utilizing the maximum-likelihood method.
